# The missing link: *Bordetella petrii *is endowed with both the metabolic versatility of environmental bacteria and virulence traits of pathogenic Bordetellae

**DOI:** 10.1186/1471-2164-9-449

**Published:** 2008-09-30

**Authors:** Roy Gross, Carlos A Guzman, Mohammed Sebaihia, Vítor AP Martins dos Santos, Dietmar H Pieper, Ralf Koebnik, Melanie Lechner, Daniela Bartels, Jens Buhrmester, Jomuna V Choudhuri, Thomas Ebensen, Lars Gaigalat, Stefanie Herrmann, Amit N Khachane, Christof Larisch, Stefanie Link, Burkhard Linke, Folker Meyer, Sascha Mormann, Diana Nakunst, Christian Rückert, Susanne Schneiker-Bekel, Kai Schulze, Frank-Jörg Vorhölter, Tetyana Yevsa, Jacquelyn T Engle, William E Goldman, Alfred Pühler, Ulf B Göbel, Alexander Goesmann, Helmut Blöcker, Olaf Kaiser, Rosa Martinez-Arias

**Affiliations:** 1 Chair of Microbiology, Biocenter, University of Würzburg, Am Hubland, D-97074 Würzburg, Germany; 2 Department of Vaccinology and Applied Microbiology, Helmholtz Center for Infection Research, D-38124 Braunschweig, Germany; 3 Wellcome Trust Sanger Institute, Wellcome Trust Genome Campus, Hinxton, Cambridge, CB10 1SA, UK; 4 Division of Molecular Biotechnology, Helmholtz Center for Infection Research, D-38124 Braunschweig, Germany; 5 Department of Microbial Pathogenesis, Helmholtz Center for Infection Research, D-38124 Braunschweig, Germany; 6 Institut de Recherche pour le Développement UMR 5096, CNRS-UP-IRD 911, Avenue Agropolis, BP 64501, 34394 Montpellier, Cedex 5, France; 7 Center for Biotechnology (CeBiTec), Bielefeld University, D-33501 Bielefeld, Germany; 8 Chair of Genetics, Bielefeld University, D-33501 Bielefeld, Germany; 9 Institute for Microbiology and Hygiene, Charité Berlin, Dorotheen-Str. 96, D-10117 Berlin, Germany; 10 Department of Molecular Microbiology, Washington University School of Medicine, St. Louis, MO 63110, USA; 11 Department of Microbiology and Immunology, University of North Carolina, Chapel Hill, USA; 12 Department of Genome Analysis, Helmholtz Center for Infection Research, D-38124 Braunschweig, Germany; 13 Mathematics and Computer Science Division, Argonne National Laboratory, USA; 14 Roche Diagnostics GmbH, Nonnenwald 2, D-82377 Penzberg, Germany; 15 BASF Plant Science GmbH, D-67117 Limburgerhof, Germany

## Abstract

**Background:**

*Bordetella petrii *is the only environmental species hitherto found among the otherwise host-restricted and pathogenic members of the genus *Bordetella*. Phylogenetically, it connects the pathogenic Bordetellae and environmental bacteria of the genera *Achromobacter *and *Alcaligenes*, which are opportunistic pathogens. *B. petrii *strains have been isolated from very different environmental niches, including river sediment, polluted soil, marine sponges and a grass root. Recently, clinical isolates associated with bone degenerative disease or cystic fibrosis have also been described.

**Results:**

In this manuscript we present the results of the analysis of the completely annotated genome sequence of the *B. petrii *strain DSMZ12804. *B. petrii *has a mosaic genome of 5,287,950 bp harboring numerous mobile genetic elements, including seven large genomic islands. Four of them are highly related to the *clc *element of *Pseudomonas knackmussii *B13, which encodes genes involved in the degradation of aromatics. Though being an environmental isolate, the sequenced *B. petrii *strain also encodes proteins related to virulence factors of the pathogenic Bordetellae, including the filamentous hemagglutinin, which is a major colonization factor of *B. pertussis*, and the master virulence regulator BvgAS. However, it lacks all known toxins of the pathogenic Bordetellae.

**Conclusion:**

The genomic analysis suggests that *B. petrii *represents an evolutionary link between free-living environmental bacteria and the host-restricted obligate pathogenic Bordetellae. Its remarkable metabolic versatility may enable *B. petrii *to thrive in very different ecological niches.

## Background

The genus *Bordetella *comprises several human and animal pathogens and nine species are currently described [see Additional file 1]. Human-restricted *B. pertussis *and *B. parapertussis *cause whooping cough, whereas *B. bronchiseptica *and *B. avium *are responsible for respiratory infections in many mammals and birds, respectively [1-3]. Despite the availability of vaccines, there are still 300,000 deaths/year caused by *B. pertussis *and significant economic losses associated with infections in poultry and cattle. The genomes of these Bordetellae were recently sequenced and analyzed [4,5]. The genome sequence of *B. avium *revealed that it is quite divergent from the mammalian pathogens. In contrast, the genomes of the human pathogens *B. pertussis *and *B. parapertussis *showed that they are independent derivatives of *B. bronchiseptica*-like ancestors. They both underwent significant gene loss, probably mediated by insertion sequence (IS) elements, during the process of host adaptation, as indicated by their smaller genome sizes compared to that of *B. bronchiseptica *and the presence of many pseudogenes. The evolution of human-adapted species is characterized by massive genome reduction, lack of horizontal acquisition of genetic material and significant differences in virulence gene expression among species [4,6].

The type strain of the only environmental species of the genus, *B. petrii*, was isolated from a dechlorinating bioreactor enriched by river sediment [7]. *B. petrii *strains were repeatedly found in environmental samples, e.g. microbial consortia degrading aromatic compounds [8,9]. Recently, two *B. petrii *strains were isolated from patients suffering from mandibular osteomyelitis and chronic suppurative mastoiditis [10,11]. Bacteria closely related to *B. petrii *were also found in cystic fibrosis patients [12]. Moreover, *B. petrii *strains were also isolated from marine sponges and a grass root consortium [13,14]. Phylogenetic analysis shows that the Bordetellae are closely related to *Achromobacter *and *Alcaligenes *[see Additional file 1] [2,7]. These environmental organisms occasionally cause severe nosocomial infections and they are also isolated from patients with cystic fibrosis [15,16]. In addition to their medical relevance, these microorganisms also have an intrinsic biotechnological interest, because they show a remarkable capacity to degrade aromatic compounds and detoxify heavy metals [17,18].

Since *B. petrii *exhibits properties of host-associated and environmental bacteria, it is important to perform a genomic analysis of this bacterium in order to understand the genetic basis of its versatility. The *B. petrii *genome provides insights into the evolutionary history of the Bordetellae, acting also as a spyglass to the *Alcaligenes*/*Achromobacter *group for which, despite their biotechnological potential, no sequenced genomes are available yet.

## Results and discussion

### Genome features and comparative analysis

**Figure 1:**
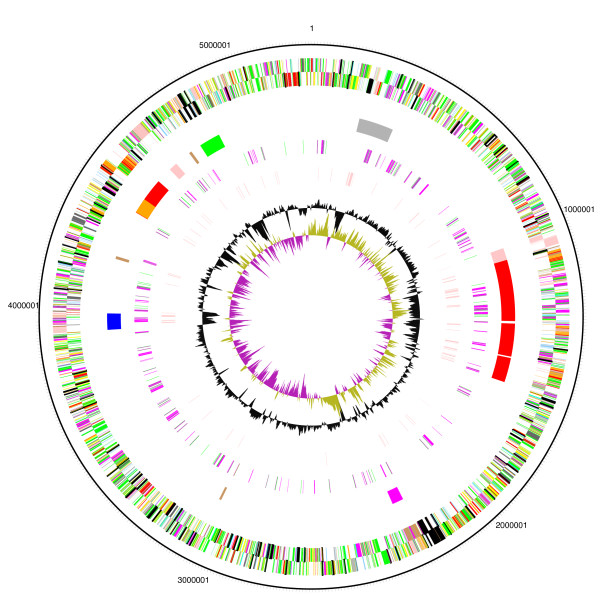
**Circular representations of the genome of *B. petrii***. The circles represent, from the outside in; 1+2, all transcribed CDS (clockwise and counter-clockwise, respectively) [Colour coding: dark blue, pathogenicity/adaptation; black, energy metabolism; red, information transfer; dark green, surface associated; cyan, degradation of large molecules; magenta, degradation of small molecules; yellow, central/intermediary metabolism; pale green, unknown; pale blue, regulators; orange, conserved hypothetical; brown, pseudogenes; pink, phage and IS elements; grey, miscellaneous]; 3, genomic islands [grey: GI; red (clockwise): GI1, 2, 3 and 6; dark purple: GI4; blue: GI5; Green: GI7; light purple: prophages; brown: remnants of prophages or GI]; 4, aromatic compounds metabolism (purple) and *bug *(green) genes, a gene family which has experienced a vast amplification in the Bordetellae possibly encoding periplasmic binding proteins [53]; 5, IS elements; 6, GC content (plotted using a 10 kb window); 8, GC deviation [(G-C)/(G+C) plotted using a 10 kb window; khaki indicates values > 1, purple < 1].

The general features of the *Bordetella *genomes are shown in Table 1 and Figure 1. The genome of *B. petrii *has a similar size (5,287,950 bp) and coding-capacity (5,034 CDSs) to that of *B. bronchiseptica *(5,339,179 bp; 5,019 CDSs). The genomes of *B. pertussis*, *B. parapertussis *and *B. avium *are much smaller, showing massive gene loss during host-adaptation. The overall GC content (65.5%) of *B. petrii *is intermediate between that of *B. bronchiseptica *(68.08%) and *B. avium *(61.58%). *B. petrii *has 125 pseudogenes, less than *B. pertussis *(358) and *B. parapertussis *(220), but more than *B. bronchiseptica *(18) and *B. avium *(68).

**Table 1:** General features of the *Bordetella *genomes

	***B. pertussis***	***B. parapertussis***	***B. bronchiseptica***	***B. avium***	***B. petrii***
Size (bp)	4,086,186	4,773,551	5,338,400	3,732,255	5,287,950
GC content (%)	67.72	68.10	68.07	61.58	65.48
Coding sequences	3,816	4,404	5,007	3,417	5,034
Pseudogenes	358	220	18	68	125
Coding density	91.6%	92.2%	92.0%	88.6%	90,6%
Average gene size (bp)	978	987	978	972	957
rRNA operons	3	3	3	3	3
tRNA	51	53	55	61	51
IS*481*	238	0	0	0	0
IS*1001*	0	22	0	0	1 (truncated)
IS*1002*	6	90	0	0	0
IS*1663*	17	0	0	0	6
IS*3*-family and others	0	0	0	0	98

**Figure 2:**
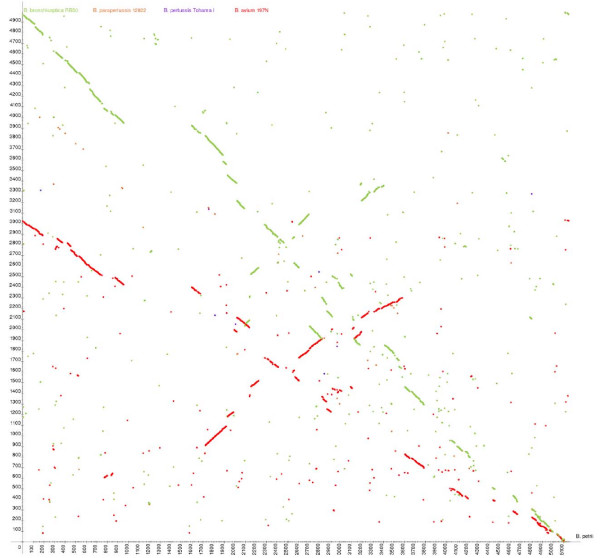
***B. petrii *syntenic plot**. Syntenic plot between the genomes of *B. petrii *strain DSMZ12804, *B. bronchiseptica *RB50, *B. parapertussis *12822, *B. pertussis *Tohama I and *B. avium *197N. The diagram depicts x-y plots of dots forming syntenic regions between *B. petrii *(x-axis) and the three other *Bordetella *genomes (y-axis), with coordinates corresponding to the CDS number in each genome. Each coloured dot (green, *B. bronchiseptica *RB50; orange, *B. parapertussis *12822; lilac, *B. pertussis *Tohama I; red, *B. avium *197N) represents a *B. petrii *strain DSMZ12804 CDS having an orthologue in one of the three compared genomes. The orthologues were identified by bi-directional best BLASTP matches of amino acid sequences (e-value < e^-30^).

The genomes of *B. petrii *and *B. avium *show lower similarity and synteny than those of mammalian-adapted Bordetellae, suggesting that they are most distantly related. Reciprocal BLASTP analysis with other sequenced Bordetellae identified coding sequences unique to *B. petrii *(Figure 2) [4,5]. *B. petrii*, *B. bronchiseptica *and *B. avium *share 2,049 CDSs, which represent the *Bordetella *core gene set, which was likely inherited from a common ancestor. *B. petrii *shares more CDSs with *B. bronchiseptica *(2,884) than with *B. avium *(2,229). Interestingly, *B. petrii *has 1,825 CDSs that are not present in either *B. bronchiseptica *or *B. avium*. A large number of these *B. petrii*-unique CDSs (1,157 CDSs and 188 genes encoding transposases) are located on mobile elements and encode accessory metabolic functions (Table 2).

**Table 2:** Major features of the *B. petrii *genomic islands

**Genomic islands (CDS)**	**Coordinates (size in bp)**	**Major features**
GI (Bpet0187-0310)	201731..346691 (144961)	No integrase and direct repeats (DR)
		Capsular polysaccharide genes
		2 autotransporters
		Metabolism of phthalate and protocatechuate, urea amidohydrolase

GI1 (Bpet1009-1275)	1084007..1339483 (255477)	*clc*-like, integrase, DR (3' end of tRNA^Gly^)
		Metabolism of an unknown aromatic compound (Bpet1116-1123)

GI2 (Bpet1288-1437)	1350143..1493539 (143397)	*clc*-like, integrase, DR (3' end of tRNA^Gly^)
		Metabolism of benzoate, benzylalcohol, 3-hydroxybenzoate, putative monooxygenase (Bpet1330)
		Putative (chloro)phenol monooxygenase (Bpet1331)
		nitrile hydratase (Bpet1415-1416)

GI3 (Bpet1438-1545)	1493557..1595653 (102110)	Almost identical to the *clc *element, integrase, DR (3' end of tRNA^Gly^)
		Metabolism of chlorocatechol, anthranilate
		Short chain dehydrogenase (Bpet1512)

GI4 (Bpet2166-2216)	2250672..2297721 (47176)	Tn*4371*-like, integrase, (T)6 DR

GI5 (Bpet3699-3770)	3912214..3979917 (67704)	Integrase, features of conjugative transposons
		Metabolism of chlorocatechol (2 gene clusters), tetrachlorobenzene
		4 glutathione S-transferase (Bpet3724-3727)

GI6 (Bpet4174-4316)	4417761..4576856 (159096)	*clc*-like, integrase, DR (3' end of tRNA^Gly^)
		Multidrug efflux pump
		Iron transport system.

GI7 (Bpet4544-4630)	4804478..4893272 (88795)	Integrase, DR (3' end of tRNA^Phe^), features of conjugative transposons
		Several heavy metal resistance systems

### Mobile genetic elements – a mosaic genome

**Figure 3:**
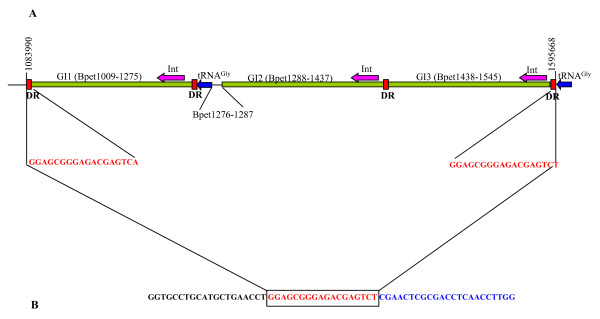
**Schematic representation of the region encompassing GI1, GI2 and GI3 in the wild type strain (A) and in a spontaneous deletion variant (B)**. The duplicated sequence at the insertion site is shown in red and marked as DR. The position of the tRNA^Gly ^genes (blue) are indicated, as well as the location of the integrase encoding genes (red arrows).

The most prominent feature that distinguishes *B. petrii *from other Bordetellae is the presence of seven laterally acquired genomic islands (GI1-GI7). The locations of these GIs on the chromosome correlate with atypical GC-content and GC-bias (Figure 1). These GIs have the characteristic features of mobile elements collectively known as integrative and conjugative elements (ICE). Four of the *B. petrii *GIs (GI1, GI2, GI3 and GI6) are related to the *clc *element of *Pseudomonas knackmussii *B13, which codes for proteins involved in the biodegradation of chloroaromatics [19]. The similarity between these four GIs and the *clc *element is mostly confined to the conjugal transfer region and varies substantially (42%–98%) between individual genes within this region [see Additional file 2]. However, among these GIs, GI3 is the most similar (78%–98%) to the *clc *element and also carries all the chloroaromatic degredation genes found in the *clc *element. Like the *clc *element in B13, these four GIs are integrated into the 3'-end of tRNA^Gly ^genes, are flanked by a 15–18 bp direct repeat generated by the duplication of the 3'-end of tRNA, and each encodes an integrase highly similar (88%, 69%, 100% and 52% amino acid identity) to that of the *clc *element (IntB13). GI1, GI2 and GI3 are located in close proximity within a 510 kb region (Figures 1, 3). The *clc*-type mobile elements are self transmissible and *B. petrii *may have obtained them from environmental Pseudomonads, with which it shares habitat [20]. During the conjugal transfer process, ICEs are excised from the chromosome and form a circular intermediate [21]. Interestingly, during in *vitro *culture the whole 510 kb region encompassing GI1, GI2 and GI3 is deleted in a fraction of the bacterial population. Sequence analysis of the deletion point revealed a regenerated 18 bp integration site (Figure 3), indicating that these GIs are still active in terms of excision from the chromosome. In fact, circular intermediates were detected for all GIs in *B. petrii *cells during normal growth (Lechner and Gross, unpublished results).

The remaining GIs (GI4, GI5 and GI7) encode a conjugal transfer system similar to the type IV secretion systems associated with plasmids. GI4 is highly similar to Tn*4371 *of *Ralstonia eutropha *A5/*Cupriavidus oxalaticus*, a 55-kb conjugative transposon that carries genes involved in the degradation of biphenyl or 4-chlorobiphenyl [22]. The features of all GIs are summarized in Table 2.

It has been previously reported that the chromosomes of *B. pertussis *and *B. parapertussis *carry a large number of IS elements (261 and 112, respectively). These IS elements are responsible for the extensive shuffling and deletions in their genomes [4]. In contrast, *B. bronchiseptica *and *B*. *avium *carry none. *B. petrii *carries 105 IS elements, most of which belong to the IS*3*-family. Interestingly, there are six copies of IS*1663 *which was found in *B. pertussis *and one truncated copy of IS*1001 *(Bpet1198) which was found in *B. parapertussis*. The vast majority (66%) of the *B*. *petrii *IS are located within GIs, whereas 34% are randomly dispersed around the chromosome. *B. petrii *also carries two intact prophages (phage 1, Bpet0942-1008; phage 2, Bpet4384-4432) (Figure 1), and phage-like particles were indeed observed by electron microscopy of the sequenced strain [7]. Phage 1 is inserted at the 3'end of a gene encoding a tRNA^cys^, while phage 2 is related to bacteriophage Mu. In addition, there are several regions on the *B. petrii *genome which may encode prophage remnants (Figure 1). In contrast, *B. avium *carries three and *B. bronchiseptica *carries four prophages, however, none of them is related to the *B. petrii *phages.

The GIs (GI1-GI7), IS elements and prophages represent 22% of the *B. petrii *genome. This highly mosaic genome structure reflects extensive DNA exchange and provides first evidence for a considerable horizontal gene transfer-driven evolution in the genus *Bordetella*.

### Metabolism of *B. petrii *– genomic basis for metabolic versatility

**Figure 4:**
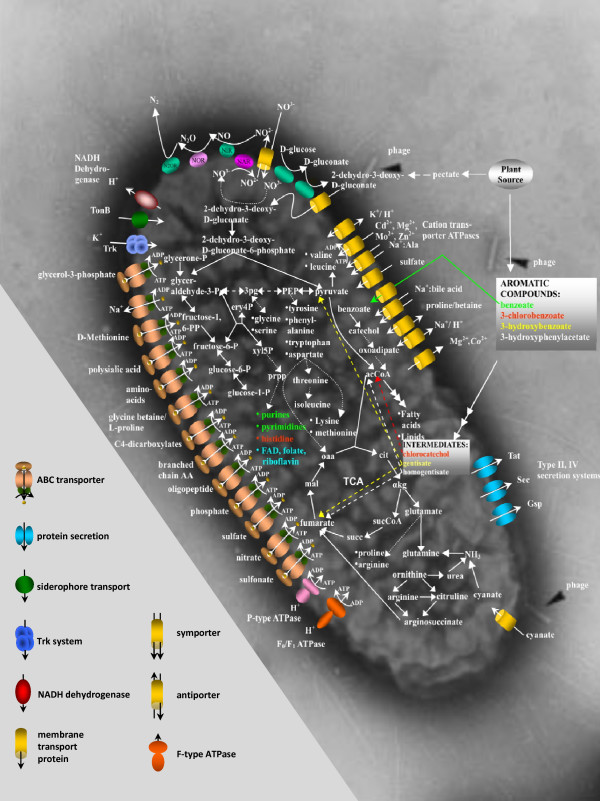
**Schematic presentation of the central intermediate metabolism of *B. petrii***. Dotted arrows indicate the presence of multiple reaction steps between two metabolites. Black triangles indicated phage particles. On the bottom left the meaning of the various symbols for membrane components are explained. The electron micrograph shows the sequenced *B. petrii *strain.

The central metabolic pathways are remarkably similar between *B. petrii *and the pathogenic Bordetellae. For instance, the glycolytic pathway is incomplete, lacking the determinants for glucokinase and phosphofructokinase. *B petrii *encodes all the enzymes needed for the TCA cycle and for the synthesis of all essential nucleotides, cofactors, amino acids and fatty acids. However, the *in silico *analysis of the genome sequence of *B. petrii *suggests the presence of a set of auxiliary pathways for the use of alternative nutrients, such as gluconate, degraded plant products, cyanate (as N-source), and various aromatic compounds (see below), which may contribute to the ability of *B. petrii *to thrive in different environments (Figure 4).

Gluconate is an intermediate in the degradation of sugar acids and sugar derivatives, which are components of more complex molecules, such as plant materials and secondary metabolites. The genome analysis of *B. petrii *suggests that this microorganism can also utilize gluconate via a variant of the Entner-Doudoroff (ED) pathway, which is only found in few bacteria, such as *Rhodobacter sphaeroides*, but absent in all the other sequenced Bordetellae [23]. In this pathway, gluconate is first transformed by a gluconate dehydratase (Bpet3300) to 2-dehydro-3-deoxy-D-gluconate, which is then imported into the cytoplasm via 2-dehydro-3-deoxy-D-gluconate permease (Bpet1985) and phosphorylated to 2-dehydro-3-deoxy-D-gluconate-6-phosphate by a 2-dehydro-3-deoxy-D-gluconate kinase (KdgK, Bpet0876). This compound is then converted to glyceraldehyde 3-phosphate and pyruvate by the action of 2-dehydro-3-deoxy-D-gluconate-6-phosphate aldolase (KdgA, Bpet0875). A IclR family transcription regulator (Bpet0874) is present immediately upstream of the *kdgA *and *kdgK *genes suggesting that this variant ED pathway may be inducible by this regulator depending on the availability of the external carbon source. In this regard, it is surprising that although a periplasmic glucose dehydrogenase (*gcd*, Bpet4644), which converts glucose to gluconate, is encoded in the genome, *B. petrii *is unable to metabolize glucose [7].

Interestingly, *B. petrii *encodes a protein (Bpet0241) with significant similarity to pectate lyases, extracellular enzymes secreted by phytopathogens, such as *Erwinia carotovora*, which catalyze the cleavage of pectate [24-26]. The reaction products are channeled into the variant ED pathway [27]. In agreement with this, the determinants for two other important enzymes of these pathways, 2-deoxy-D-gluconate 3-dehydrogenase (Bpet0046, Bpet1132) and altronate hydrolase (Bpet0414) are present, indicating that *B. petrii *uses plant cell-wall constituents as nutrient sources, which is in line with the isolation of a *B. petrii *strain from a plant root consortium [14]. The presence of a cyanate transporter (Bpet3878) and a cyanate lyase (Bpet3621) catalyzing the decomposition of cyanate into ammonia and bicarbonate, suggests that *B. petrii *is not only able to cope with the toxicity of environmental cyanate, but like *E. coli *may use it as a nitrogen source [28]. A striking difference between *B. petrii *and the other Bordetellae is that it has a facultative anaerobic energy metabolism and can utilize nitrate as the terminal electron acceptor during anaerobic respiration [7]. Accordingly, on its core genome *B. petrii *encodes the factors (nitrate-, nitrite-, and nitrous oxide reductases) necessary to carry out complete denitrification from nitrate to N_2_. Interestingly, only some of the respective genes are present in the pathogenic Bordetellae. *B. bronchiseptica *appears to encode a functional nitrate reductase, while *B. parapertussis *has a truncated *napD *gene probably leading to a non-functional nitrate reductase, and *B. pertussis *does not encode genes involved in denitrification at all.

### Metabolic talents of *B. petrii *– degradation of aromatic compounds

**Figure 5:**
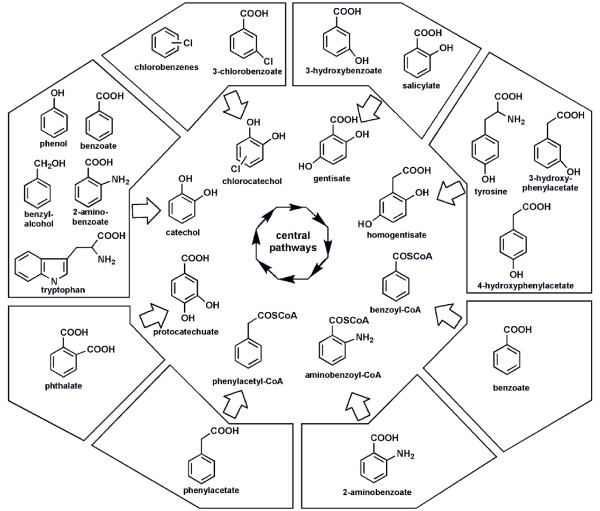
**Overview of the degradation capacity of aromatic compounds by *B. petrii***. Aromatic compounds (boxed) are funneled through a variety of peripheral reactions (represented by arrows) into central intermediates, which are then processed by a central pathway to TCA cycle intermediates. However, neither salicylate nor phenylacetate could be used by *B. petrii *as sole source of carbon and energy (data not shown), probably due to the absence of a ferredoxin reductase encoding gene within the salicylate 5-hydroxylase encoding gene cluster (Bpet2804-2806 cluster), and an incomplete *paaZ *gene in the phenylacetate catabolic gene cluster (Bpet 1923–1935). 4-hydroxyphenylacetate is used as sole source of carbon and energy (data not shown), even though no gene with similarity to those encoding 4-hydroxyphenylacetate 3-hydroxylase was found in the genome (see Methods for experimental details).

The *in silico *analysis of the *B. petrii *genome revealed an unusual metabolic capability regarding the degradation of aromatic compounds summarized in Figure 5. For the metabolisation of some of these compounds including benzoate, phthalate, 3-hydroxybenzoate, 3-hydroxyphenylacetate and 4-hydroxyphenylacetate we have obtained experimental evidence (data not shown).

A prominent feature is the presence of a surprisingly large number of genes coding for enzymes of the chloroaromatic metabolism. One complete set of chlorocatechol pathway genes (Bpet1533-1538) is localized on GI3 highly related to the *clc *element of *Pseudomonas knackmussii *B13. A second set of chlorocatechol pathway genes (Bpet3747-3752) is located on GI5 and shows high similarity to genes identified in the 1,2,4-trichlorobenzene degrading *Pseudomonas *strain P51 [29]. A third possible chlorocatechol gene cluster encoding proteins only distantly related to those previously characterized is also located on GI5. This multiplicity of chlorocatechol gene clusters probably endows this strain with the ability to effectively metabolize a broad range of differently substituted chlorocatechols, providing thereby a valuable competitive advantage to thrive in polluted environments [30-32].

On GI5, chlorocatechol genes are preceded by a gene cluster (Bpet3738-3742) encoding a chlorobenzene dioxygenase and chlorobenzene dihydrodiol dehydrogenase, highly homologous to those of the 1,2,4-trichlorobenzene degraders *Cupriavidus *strain PS12 and *Pseudomonas *strain P51 [33,34]. The absence of any linked extradiol dioxygenase encoding gene suggests that similarly to what is observed in the strains PS12 and P51, this cluster has specifically evolved for the transformation of chlorobenzenes [29,30,35].

*B. petrii *also harbors four different central pathways for aromatic metabolism via dihydroxylated central intermediates, the gentisate (Bpet0516-0517 and Bpet1429-1430, the latter located on GI2), the homogentisate (Bpet 4016–4017) and the catechol and protocatechuate branch of the 3-oxoadipate pathway (see below). Such versatility is similar to that observed in bacteria well known for their high metabolic capabilities, such as *P. putida *KT2440 and *Cupriavidus necator *[36]. Only genes encoding enzymes of the homogentisate pathway were detected in all other sequenced Bordetellae, and those involved in the gentisate pathway are only present in *B. avium*.

Various natural aromatic compounds are known to be degraded via either the catechol or the protocatechuate branch of the 3-oxoadipate pathway, which thus plays a key role in bacterial aromatic catabolism. This pathway is widespread in *Proteobacteria*, however, all previously sequenced *Bordetella *strains are devoid of complete pathways and only a *catIJCD *cluster (Bpet4528-4532) is present as a remnant. In *B. petrii*, complete pathways are formed by genes of the protocatechuate branch (Bpet0260-0254) located in a genomic region with a quite low GC content, but lacking other typical features of a genomic island (named GI in Table 2 and in Additional file 3), and two clusters localized on GI2, encoding the enzymes of the catechol branch. We believe this to be the first example of natural horizontal transfer of this central aromatic metabolic pathway. Notably, also peripheral pathways for funneling benzoate (Bpet1396-1399) and benzylalcohol (Bpet1387-1388) to catechol are located on GI2. A peripheral pathway for channeling phthalate to protocatechuate (Bpet0248-0252) is located on GI1. The low number of peripheral routes contrasts with the situation in other *Proteobacteria*, such as *B. xenovorans *LB400, where five substrates are degraded via protocatechuate [37].

A strategy of aerobic degradation of aromatics alternative to the metabolism via dihydroxylated intermediates is initiated by CoA ligases [38,39]. *B. petrii *carries gene clusters required for the metabolism of all three substrates, which are known to be degraded by such a strategy (Bpet0549-0554 for anthranilate metabolism and Bpet1924-1935 for phenylacetate metabolism, which are present in other Bordetellae, and Bpet3568-3575 for benzoate metabolism). As degradation via CoA derivatives needs less oxygen molecules compared to degradation via dihydroxylated intermediates, this may provide *B. petrii *with a selective advantage to also thrive in oxygen limited environments. For an overview about the aromatic degradation capabilities of *B. petrii *see Figure 5 and Additional file 3.

### "Virulence factors" of *B. petrii *and host-restricted Bordetellae

Pathogenic Bordetellae produce many surface associated or secreted virulence factors [1]. *B. petrii *does not code for the well-characterized toxins of classical Bordetellae (i.e., pertussis toxin, adenylate cyclase toxin and dermonecrotic toxin). However, it produces a low amount (22–53 nM/OD) of the non-protein tracheal cytotoxin (TCT), a spontaneously released muramylpeptide that disrupts epithelia [40]. This expression range is comparable to the expression levels observed for the broad host range pathogen *B. bronchiseptica*.

Autotransporter or type V secretion proteins play a major role in *Bordetella *virulence [1]. *B. bronchiseptica *encodes 21 autotransporter proteins, whereas *B. petrii *encodes only four (Bpet0190, Bpet0193, Bpet3980, Bpet4156) [4]. Two of them (Bpet0190 and Bpet0193) are paralogous and show significant homology to the putative serine protease autotransporter BB2301 of *B. bronchiseptica*, whereas the other two do not show significant similarity to any database entry. Pathogenic Bordetellae also encode a two-partner secretion system which allows secretion of the colonization factor filamentous hemagglutinin (FhaB) [41]. This protein requires the FhaC protein for translocation across the outer membrane. *B*. *petrii *encodes proteins related to FhaB and FhaC (Bpet600-601). *B. petrii *FhaB has 3039 amino acids and its N-terminus, which is required for export, is well-conserved with respect to pathogenic Bordetellae. However, the overall similarity with FhaB of pathogenic Bordetellae is very low, being more similar to putative secreted proteins of plant pathogens (e.g., *Ralstonia*, *Xylella *and *Burkholderia*). This suggests that the *fha *genes found in *B. petrii *and the pathogenic Bordetellae may not be orthologous.

Other protein secretion systems are differentially distributed among Bordetellae. In contrast to the mammalian pathogens, *B. petrii *and *B*. *avium *lack type I and III protein secretion systems, but encode a type II secretion system (Bpet2855-2866 and BAV0331-0343). Type IV secretion systems are either engaged in protein transport to the extracellular medium or into eukaryotic cells, or they are involved in DNA-transfer. There are three gene clusters in *B. petrii*, which are associated with GIs and appear to encode complete type IV secretion systems (Bpet2204-2215, Bpet3701-3719, Bpet4615-4626). None of these systems appears to be related to the pertussis toxin exporting type IV system of *B. pertussis*, being probably involved in conjugal DNA transfer.

The *B. petrii *genome carries three fimbrial gene clusters (Bpet0111-0114, Bpet4129-4132 and Bpet4464-4468), each encoding a major fimbrial subunit, a fimbrial assembly chaperone, an outer membrane usher protein and a fimbrial adhesin. The chaperone and usher proteins have significant sequence similarities with their counterparts in the pathogenic Bordetellae, with e-values ranging from 6e-23 to 3e-35 for the chaperones and e-values ranging from 3e-71 to 6e-103 for the usher proteins. In contrast, the homology of the major structural proteins and fimbrial adhesins with the proteins of the pathogenic Bordetellae is very low (e-values > 1e-5). Similarly to *B*. *avium, B*. *petrii *lacks the genes for the biosynthesis of siderophores. However, it specifies 12 TonB-dependent iron scavenging receptors, which may be important for iron acquisition in soil and plant-associated environments.

Most virulence factors are coordinately regulated in pathogenic Bordetellae by a single master regulator, the BvgAS two-component system [42,43]. This system consists of two proteins, a response regulator (BvgA) and a multidomain histidine kinase (BvgS), consisting of a periplasmic solute-binding domain, and, on the cytoplasmic side, of a PAS, a transmitter, a receiver and an HPt domain. Although the *B*. *petrii *BvgA (Bpet 4471) is highly homologous to the BvgA proteins of the pathogenic Bordetellae (65% amino acid similarity), the structure of the *B*. *petrii *equivalents of BvgS is more complex. First of all, there are two histidine kinases, BvgS1 (Bpet4469) and BvgS2 (Bpet4472), with significant sequence similarities to BvgS. BvgS1 contains a PAS, a transmitter and a receiver domain, whereas BvgS2 contains a transmitter and a receiver domain. In contrast to BvgS, both *B. petrii *proteins are devoid of a large periplasmic domain. The HPt domain, which in pathogenic Bordetellae is part of the multidomain BvgS protein, in *B. petrii *is an independent protein (Bpet4470) (see Additional file 4. To see legends for Additional files 1, 2 and 4 please see Additional file 5 ). This suggests that during their evolution to pathogens, the virulence regulatory system has been streamlined by the omission of one histidine kinase and incorporation of the HPt domain into the multidomain histidine kinase BvgS.

Therefore, *B. petrii *is endowed with several putative virulence factors for plant and animal hosts, such as adhesins (e.g., Fha, fimbriae) and a master virulence regulator (Bvg), but lacks *Bordetella *toxins. This suggests that pathogenic Bordetellae might have acquired their toxin genes horizontally, while adapting to an obligate pathogenic lifestyle. Alternatively, it is possible that toxin genes have been lost from the *B. petrii *lineage.

## Conclusion

The five sequenced *Bordetella *species, *B. bronchiseptica*, *B. pertussis*, *B. parapertussis*, *B. avium *and *B. petrii*, represent different states of environmental adaptation. These five organisms have followed different evolutionary paths. Evolution of the *B. bronchiseptica*-derived host restricted-pathogens *B. pertussis *and *B. parapertussis *was dominated by substantial gene decay and loss. This might also hold true for *B. avium*, which is characterized by a relatively small genome containing 68 pseudogenes. In contrast, the evolution of the environmental isolate *B*. *petrii *was dominated by horizontal acquisition of large genomic islands expanding its metabolic capacities. *B*. *bronchiseptica*, with a broad host range and the potential to survive in the environment, might represent an intermediate evolutionary state. If the Bordetellae derive from a common environmental ancestor, *B. petrii *appears to be more closely related to this ancestor than the pathogenic Bordetellae, since it is the only true environmental isolate within the genus, which is also found in association with host organisms. Moreover, *B. petrii *is the only facultative anaerobic *Bordetella *species [7]. Since closely related *Achromobacter *species are also facultative anaerobes, the common ancestor of the Bordetellae probably had a facultative anaerobic energy metabolism. During adaptation to an obligate host association, the genes for several metabolic functions, e.g. those required for denitrification, may have been deleted from the genomes of pathogenic Bordetellae, leaving just a few remnants in the various species.

## Methods

### Whole genome shotgun sequencing

For the shotgun phase we produced 103,308 paired-end sequences derived from three pTZ18R genomic libraries (with insert sizes of 1 kb, 3 kb and 10 kb) yielding 11.72-fold coverage, using dye terminator chemistry on ABI377 and MegaBACE 1000 automated sequencers. As a scaffold we used 2,688 paired-end sequences pCC1BAC libraries with insert sizes of 17–30 kb and 40–70 kb (leading to a total coverage of 5.14-fold). Assembly of the paired-end sequences was performed by PHRAP (P. Green, unpublished data; ). The complete genome sequence was finished by using the Staden package (GAP v4.8b1) [44]. Another 5,154 sequencing reads were generated to close the remaining gaps and to polish the sequence.

### Genome analysis and annotation

Curation and annotation of the genome was done using the genome annotation system GenDB [45]. A combined gene prediction strategy was applied on the assembled sequences using GLIMMER and CRITICA [46]. Putative ribosomal binding sites and transfer RNA genes were identified with RBSFINDER and tRNAscan-SE, respectively [47,48]. In a first step, automatic annotation was computed based on various tools: similarity searches were performed against different databases including SWISS-PROT, TrEMBL, KEGG, Pfam, TIGRFAM and InterPro. Furthermore, SignalP, helix-turn-helix and TMHMM were applied. Additionally, each gene was functionally classified by assigning of a Cluster of Orthologous Groups (COG) number and its corresponding COG category and Gene Ontology numbers [49,50]. Finally, the annotation of each predicted gene was manually inspected and adjusted if necessary.

### Genomic comparisons

For comparative analyses, the annotated genome sequences of the following bacteria were imported into the genome annotation system GenDB [45]: *Bordetella avium *197N (gb;AM167904), *Bordetella pertussis *Tohama I (gb;NC_002929), *Bordetella parapertussis *12822 (gb;NC_002928), *Bordetella bronchiseptica *RB50 (gb;NC_002927). Similarity searches were conducted against the genomes on the nucleotide and amino acid sequence level by using BLASTN and BLASTP, respectively [51]. Synteny plots were created with GenDB [45]. For the determination of the core genome common to all sequenced Bordetellae all the CDS were compared to each other reciprocally using BLASTP with an e-value < 1e-20 as a cut-off.

### Detection of regions with atypical GC content

Genomic regions with atypical GC content were identified using the 'sliding window' technique with a window size of 3,000 bp and a step size of 1,000 bp. For this purpose, the GC content was assumed to follow a Gaussian distribution and regions with at least 2.0 standard deviation differences from the mean were determined.

### Use of aromatic compounds as sole carbon source by *B. petrii*

Growth experiments were carried out in a minimal medium containing 10% salt solution (2.5 g NaCl, 0.5 g KH_2_PO_4_, 0.2 g KCl, 0.1 g MgCl_2 _× H_2_O, 1.5 g Tris/HCl resuspended in 100 ml distilled water), 1.8 μM CaCl_2_, 36 μM FeSO_4 _× H_2_O, 2 mM (NH_4_)SO_4_. Aromatic carbon sources were added to final concentrations of 2–5 mM each.

### Determination of TCT production

Tracheal Cytotoxin (TCT) was quantified as previously described [52].

### Accession number

The complete, annotated genome sequence has been submitted to EBI under the accession number AM902716.

## Authors' contributions

RG and CAG organized and coordinated the work and drafted the manuscript. MS, VMdS, DHP and RK performed the bioinformatic sequence analysis of the genome and participated in manuscript preparation. ML performed molecular genetic experiments to characterize the genomic islands. DB, JB, JVC, TE, LG, SH, ANK, CL, SL, BH, SM, DN, CR, SSB, KS, FJV, and TY annotated the genome sequence. FM, OK and AG annotated the sequence and coordinated annotation, JTE and WEG quantified TCT production, DHP performed growth experiments with various carbon sources, HB and RMA coordinated and performed the genomic sequencing, AP and UBG coordinated sequence analysis and participated in manuscript preparation.

## Supplementary Material

Additional file 1 Dendrogramm of the genera *Bordetella*, *Achromobacter *and *Alcaligenes*. Click here for file

Additional file 2 Comparison of four *B. petrii *GIs and the *clc *element of *Pseudomonas*. Click here for file

Additional file 3 Additional Table 1. List of genes involved in the degradation of aromatic compounds. Click here for file

Additional file 4 Schematic presentation of the Bvg-systems of *B. pertussis *and *B. petrii*. Click here for file

Additional file 5 Legends of the additional figures. Click here for file
