# Supplementary figures and images for: The missing link: Bordetella petrii is endowed with both the metabolic versatility of environmental bacteria and virulence traits of pathogenic Bordetellae
Source: BMC Genomics. 2008 Sep 30;9:449. doi: 10.1186/1471-2164-9-449 (PMC2572626; doi:10.1186/1471-2164-9-449)

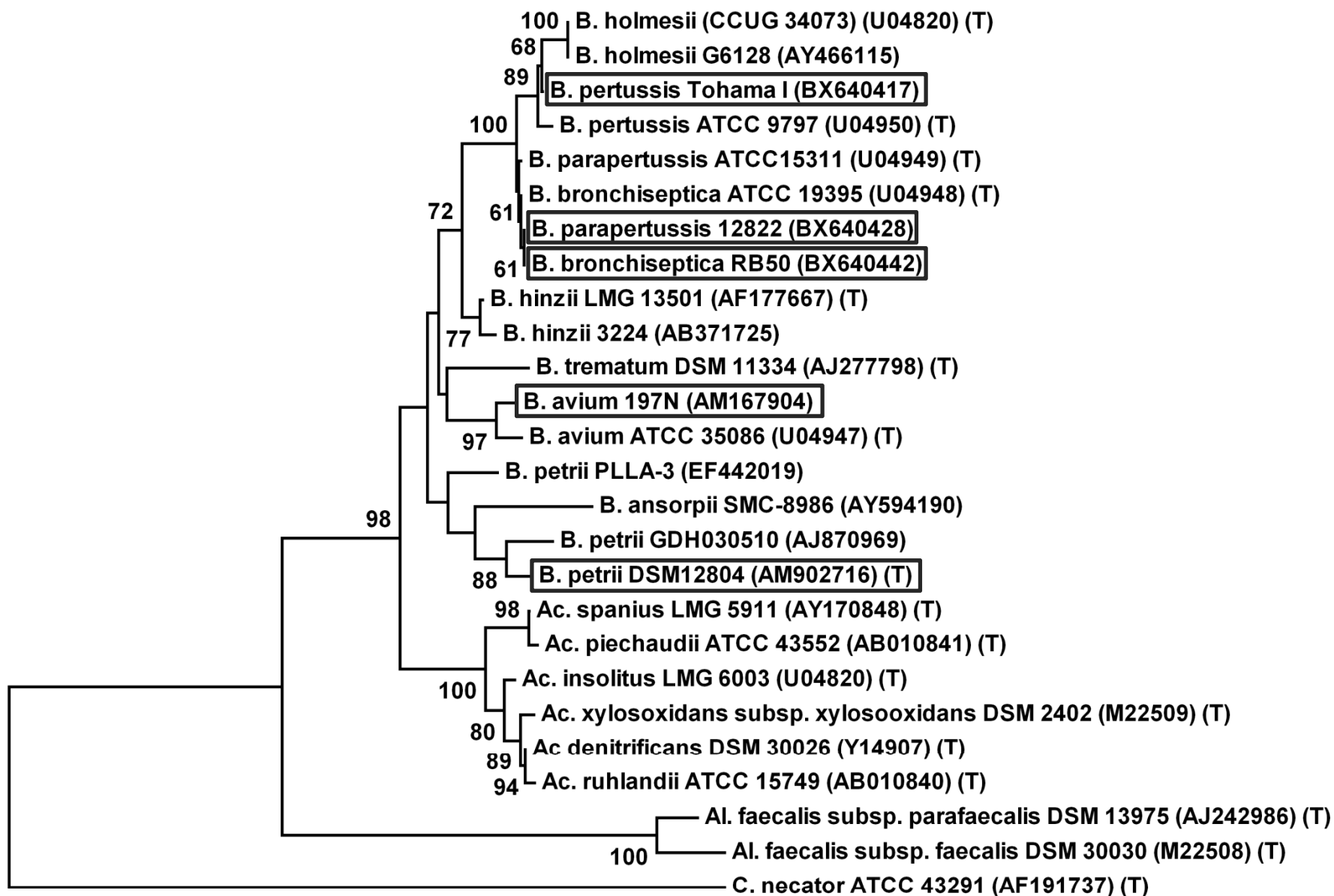

0.01

Supplement: Additional file 1 — Dendrogramm of the genera Bordetella, Achromobacter and Alcaligenes. [file 1471-2164-9-449-S1.pdf]

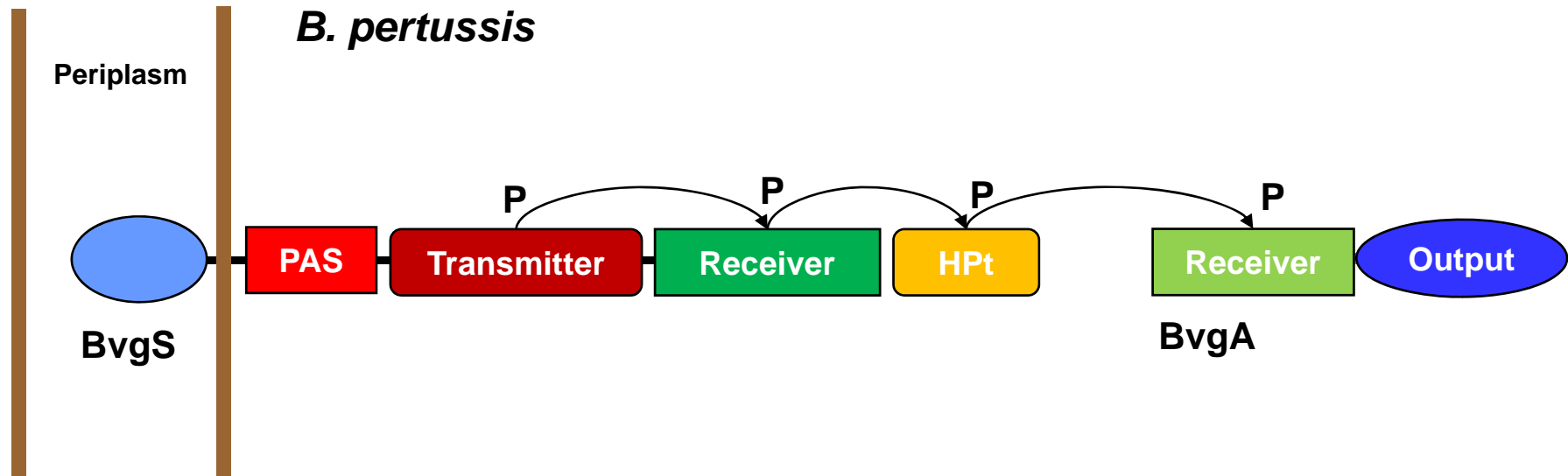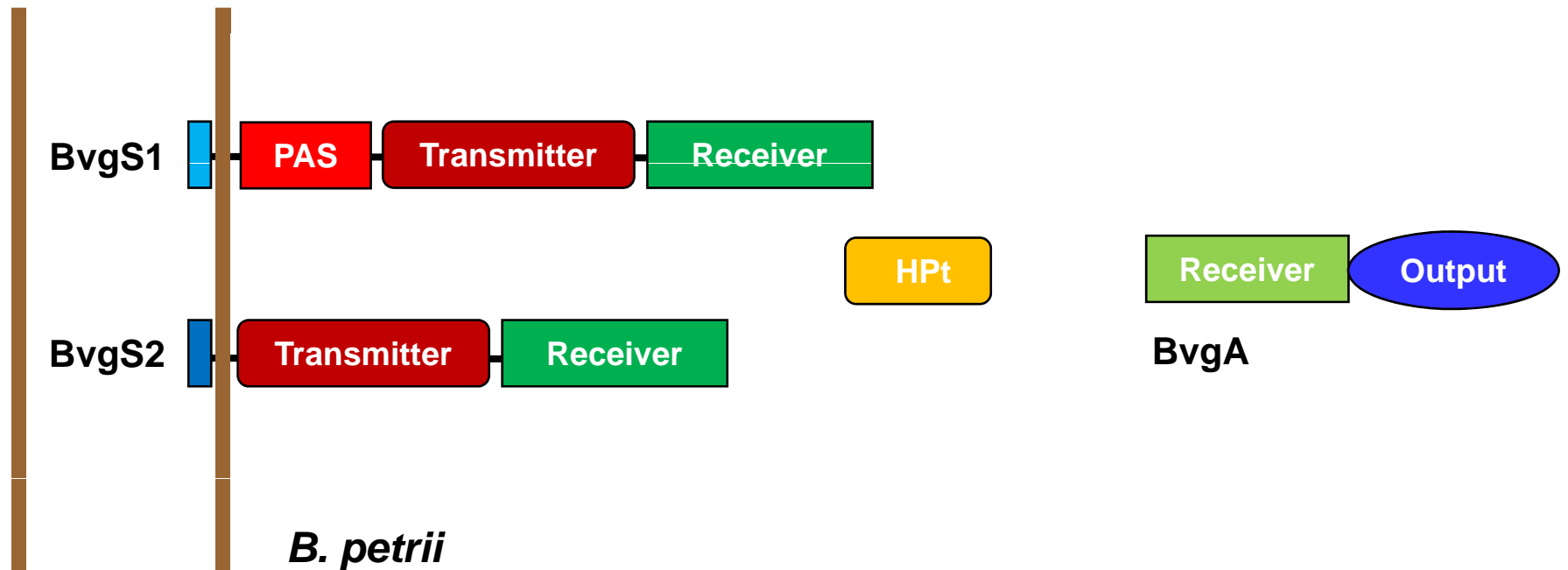

Supplement: Additional file 4 — Schematic presentation of the Bvg-systems of B. pertussis and B. petrii. [file 1471-2164-9-449-S4.pdf]
