# Supplementary material for: The missing link: Bordetella petrii is endowed with both the metabolic versatility of environmental bacteria and virulence traits of pathogenic Bordetellae
Source: BMC Genomics. 2008 Sep 30;9:449. doi: 10.1186/1471-2164-9-449 (PMC2572626; doi:10.1186/1471-2164-9-449)
Supplement: Additional file 2 — Comparison of four B. petrii GIs and the clc element of Pseudomonas. [file 1471-2164-9-449-S2.pdf]

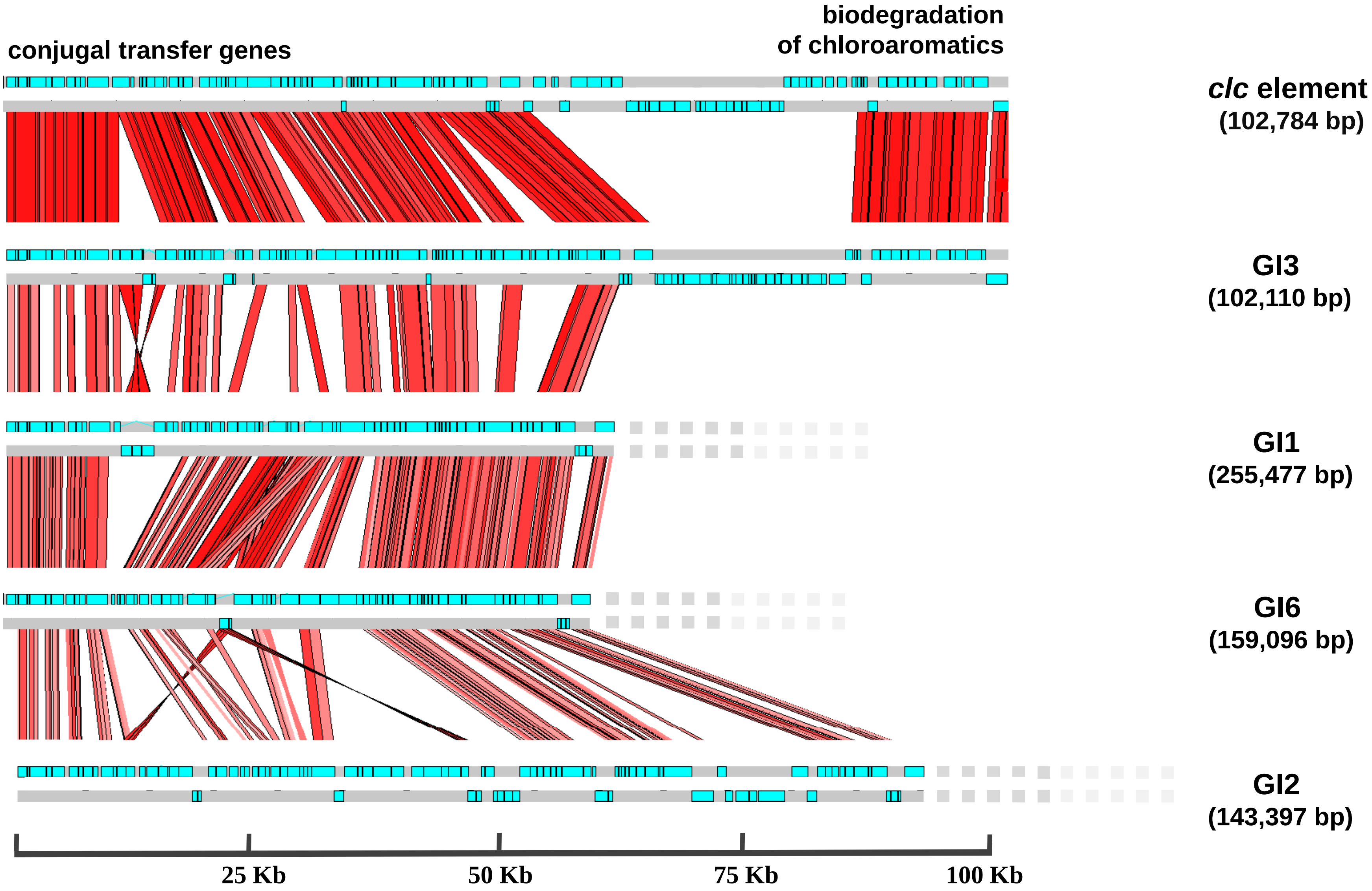
conjugal transfer genes
biodegradation
of chloroaromatics
clc element
(102,784 bp)
GI3
(102,110 bp)
GI1
(255,477 bp)
GI6
(159,096 bp)
GI2
(143,397 bp)
25 Kb
50 Kb
75 Kb
100 Kb
