# Supplementary material for: The missing link: Bordetella petrii is endowed with both the metabolic versatility of environmental bacteria and virulence traits of pathogenic Bordetellae
Source: BMC Genomics. 2008 Sep 30;9:449. doi: 10.1186/1471-2164-9-449 (PMC2572626; doi:10.1186/1471-2164-9-449)
Supplement: Additional file 3 — Additional Table 1. List of genes involved in the degradation of aromatic compounds. [file 1471-2164-9-449-S3.doc]

**Additional table 1.** List of genes involved in the degradation of aromatic compounds

| **Gene** | **Location**  **on GI** | **Product** | **Substrate** | **Pathway type** |
| --- | --- | --- | --- | --- |
| Bpet0248 | GI | MarR-family transcriptional regulator | phthalate | peripheral |
| Bpet0249 *pht4* | GI | 4,5-dihydro-4,5-dihydroxyphthalate dehydrogenase |
| Bpet0250 *pht2* | GI | phthalate dioxygenase reductase |
| Bpet0251 *pht5* | GI | 4,5-dihydroxyphthalate decarboxylase |
| Bpet0252 *pht3* | GI | phthalate dioxygenase oxygenase |
| Bpet0253 *bug5* | GI | solute receptor |
| Bpet0254 *pcaG* | GI | protocatechuate 3,4-dioxygenase, alpha subunit | protocatechuate | central |
| Bpet0255 *pcaH* | GI | protocatechuate 3,4-dioxygenase, beta subunit |
| Bpet0256 *pcaQ* | GI | LysR-family transcriptional regulator |
| Bpet0257 *pcaC* | GI | 4-carboxymuconolactone decarboxylase |
| Bpet0258 *pcaB* | GI | 3-carboxy-cis,cis-muconate cycloisomerase |
| Bpet0259 *bug6* | GI | solute receptor |
| Bpet0260 | GI | LysR-family transcriptional regulator |
|  |  |  |  |  |
| Bpet0304 *mhaA* | GI | 3-hydroxyphenylacetate hydroxylase | 3-hydroxyphenylacetate | peripheral |
|  |  |  |  |  |
| Bpet0509 *mbhM* | - | 3-hydroxybenzoate 6-hydroxylase | 3-hydroxybenzoate | peripheral |
| Bpet0510 | - | FAD-dependent oxidoreductase |
| Bpet0511 *bug15* | - | solute receptor |
| Bpet0512 | - | putative chorismate mutase |
| Bpet0513 *mhbI* | - | maleylpyruvate isomerase | gentisate | central |
| Bpet0514 *bug16* | - | solute receptor |
| Bpet0515 | - | FAD dependent monooxygenase |
| Bpet0516 *mhbH* | - | fumarylpyruvate hydrolase |
| Bpet0517 *mhbD* | - | gentisate 1,2-dioxygenase |
| Bpet0518 | - | LysR-family transcriptional regulator |
|  |  |  |  |  |
| Bpet0549 *abmA* | - | 2-aminobenzoyl-CoA monooxygenase/reductase | 2-aminobenzoyl-CoA | central |
| Bpet0550 *abmB* | - | putative beta-hydroxyacyl-CoA dehydrogenase |
| Bpet0551 *abmC* | - | putative enoyl-CoA hydratase/isomerase |
| Bpet0552 *abmD* | - | putative acyl-CoA dehydrogenase |
| Bpet0553 *abmG* | - | 2-aminobenzoate CoA ligase | 2-aminobenzoate | peripheral |
| Bpet0554 | - | thioesterase family protein |  |  |
| Bpet0555 abmE | - | hypothetical protein |
|  |  |  |  |  |
| Bpet0681 | - | 4-hydroxyphenylpyruvate dioxygenase | tyrosine | peripheral |
|  |  |  |  |  |
| Bpet1330 | GI2 | putative monooxygenase | unknown | peripheral |
| Bpet1331 | GI2 | putative (chloro)phenol monooxygenase | unknown | peripheral |
| Bpet1332 | GI2 | TetR-family transcriptional regulator |  |  |
|  |  |  |  |  |
| Bpet1385 *benE* | GI2 | benzoate:H+ symporter family protein | benzylalcohol | peripheral |
| Bpet1386 *pcaK* | GI2 | aromatic acid:H+ symporter family protein |
| Bpet1387 *xylB* | GI2 | benzylalcohol dehydrogenase |
| Bpet1388 *xylC* | GI2 | benzaldehyde dehydrogenase |
|  |  |  |  |  |
| Bpet1389 *bug35A* (pseudo) | GI2 | solute receptor | catechol | central |
| Bpet1390 *catC* | GI2 | muconolactone isomerase |
| Bpet1391 *catD* | GI2 | 3-oxoadipate enol-lactone hydrolasease |
| Bpet1392 *catF* (pseudo) | GI2 | 3-oxoadipyl CoA thiolase |
| Bpet1393 *catI* (pseudo) | GI2 | 3-oxoadipate CoA-transferase subunit A |
|  |  |  |  |  |
| Bpet1394 *benk* | GI2 | aromatic acid:H+ symporter family protein |  |  |
| Bpet1395 | GI2 | hypothetical protein | benzoate | peripheral |
| Bpet1396 *benD* | GI2 | cis-1,2-dihydroxycyclohexa-3,5-diene-1-carboxylate dehydrogenase |
| Bpet1397 *benC* | GI2 | benzoate 1,2-dioxygenase electron transfer subunit |
| Bpet1398 *benB* | GI2 | benzoate 1,2-dioxygenase beta-subunit |
| Bpet1399 *benA* | GI2 | benzoate 1,2-dioxygenase alpha subunit |
|  |  |  |  |  |
| Bpet1400 *catA* | GI2 | catechol 1,2-dioxygenase | catechol | central |
| Bpet1401 *catB* | GI2 | muconate cycloisomerase |
| Bpet1402 *catR* | GI2 | LysR-family transcriptional regulator |
|  |  |  |  |  |
| Bpet1427 | GI2 | IclR-family transcriptional regulator |  |  |
| Bpet1428 *xlnD* | GI2 | 3-hydroxybenzoate 6-hydroxylase | 3-hydroxybenzoate | peripheral |
| Bpet1429 *hbzF* | GI2 | putative maleylpyruvate isomerase | gentisate | central |
| Bpet1430 *xlnE* | GI2 | gentisate 1,2-dioxygenase |
| Bpet1431 | GI2 | aromatic acid:H+ symporter family protein |
|  |  |  |  |  |
| Bpet1512 | GI3 | short chain dehydrogenase |  |  |
| Bpet1513 | GI3 | aromatic ring-hydroxylating dioxygenase small subunit |  |  |
| Bpet1513A (pseudo) | GI3 | aromatic ring-hydroxylating dioxygenase, large subunit |  |  |
|  |  |  |  |  |
| Bpet1533 *clcR* | GI3 | LysR family transcriptional regulator | chlorocatechol | central |
| Bpet1534 *clcA* | GI3 | chlorocatechol 1,2-dioxygenase |
| Bpet1535 *clcB* | GI3 | chloromuconate cycloisomerase |
| Bpet1536 *bug36* | GI3 | solute receptor |
| Bpet1537 *clcD* | GI3 | dienelactone hydrolase |
| Bpet1538 *clcE* | GI3 | maleylacetate reductase |
| Bpet1540 | GI3 | aromatic ring-hydroxylating dioxygenase small subunit | unknown | peripheral |
| Bpet1541 | GI3 | aromatic ring-hydroxylating dioxygenase, large subunit |
| Bpet1542 | GI3 | oxidoreductase |
| Bpet1543 | GI3 | aromatic acid:H+ symporter family protein |
|  |  |  |  |  |
| Bpet1634 | - | LysR-family transcriptional regulator |  |  |
| Bpet1635 *pcaJ2* | - | 3-oxoadipate CoA-transferase subunit B | protocatechuate/catechol | central |
| Bpet1636 *pcaI2* | - | 3-oxoadipate CoA-transferase subunit A |
| Bpet1637 | - | thiolase |
|  |  |  |  |  |
| Bpet1923 *paaX* | - | transcriptional regulator | phenylacetyl-CoA | central |
| Bpet1924 *paaA* | - | ring-oxidation complex protein 1 |
| Bpet1925 *paaB* | - | ring-oxidation complex protein 2 |
| Bpet1926 *paaC* | - | ring-oxidation complex protein 3 |
| Bpet1927 *pcaD* | - | ring-oxidation complex protein 4 |
| Bpet1928 *paaE* | - | ring-oxidation complex protein 5 |
| Bpet1929 *paaZ* (pseudo) | - | ring-opening enzyme |
| Bpet1933 *paaG* | - | enoyl-CoA hydratase |
| Bpet1934 *paaI* | - | thioesterase |
| Bpet1935 *paaK* | - | phenylacetyl-CoA ligase | phenylacetate | peripheral |
| Bpet1936 | - | acyl-CoA dehydrogenase |  |  |
| Bpet2634 *hpaH* (pseudo) | - | 2-oxo-hept-3-ene-1,7-dioate hydratase | homoprotocatechuate | central |
| Bpet2635 *hpaI* | - | 2,4-dihydroxyhept-2-ene-1,7-dioate aldolase |
|  |  |  |  |  |
| Bpet2665 *paaE*/*catF* | - | beta-ketoadipyl CoA thiolase | protocatechuate/catechol | central |
|  |  |  |  |  |
| Bpet2781 *tyrB* | - | aspartate/tyrosine/aromatic aminotransferase | tyrosine | peripheral |
|  |  |  |  |  |
| Bpet2803 *ndsR* | - | LysR-family transcriptional regulator | salicylate | peripheral |
| Bpet2804 *ndsB* | - | oxygenase large subunit of salicylate 5-hydroxylase |
| Bpet2805 *ndsC* | - | oxygenase small subunit of salicylate 5-hydroxylase |
| Bpet2806 *ndsD* | - | ferredoxin component of salicylate 5-hydroxylase |
|  |  |  |  |  |
| Bpet3150 *kynB* | - | kynurenine formamidase | tryptophan | peripheral |
| Bpet3151 *kynA* | - | tryptophan 2,3-dioxygenase |  |  |
|  |  |  |  |  |
| Bpet3348 *catR2* | - | LysR-family transcriptional regulator | catechol | central |
| Bpet3349 *catB* | - | muconate cycloisomerase |
| Bpet3350 *bug66* | - | solute receptor |
|  |  |  |  |  |
| Bpet3568 *boxA* | - | benzoyl-CoA oxygenase component A | benzoyl-CoA | central |
| Bpet3569 *boxB* | - | benzoyl-CoA oxygenase component B |
| Bpet3570 *boxC* | - | benzoyl-CoA-dihydrodiol lyase |
| Bpet3571 *boxR* | - | transcriptional regulator |
| Bpet3572 | - | unknown function |
| Bpet3573 boxD | - | 3,4-dehydroadipyl-CoA semialdehyde dehydrogenase |
| Bpet3574 *bzdA* | - | benzoate CoA-ligase | benzoate | peripheral |
| Bpet3575 | - | Lactonase | benzoyl-CoA | central |
|  |  |  |  |  |
| Bpet3588 | - | LysR-family transcriptional regulator | protocatechuate/catechol | central |
| Bpet3589 *bug74* | - | solute receptor |
| Bpet3590 *pcaI3* | - | 3-oxoadipate CoA-transferase subunit A |
| Bpet3591 *pcaJ3* | - | 3-oxoadipate CoA-transferase subunit B |
| Bpet3592 *pcaR* | - | transcriptional regulator |
|  |  |  |  |  |
| Bpet3730 *tfdF* | GI5 | maleylacetate reductase | chlorocatechol | central |
| Bpet3731 *tfdD* (pseudo) | GI5 | chloromuconate cycloisomerase |
| Bpet3735 tfdE | GI5 | dienelactone hydrolase |
| Bpet3737 *tecF* | GI5 | 2-hydroxy-6-oxo-2,4-heptadienoate hydrolase | methylcatechol |  |
|  |  |  |  |  |
| Bpet3738 *tcbA*a | GI5 | chlorobenzene dioxygenase alpha-subunit | tetrachlorobenzene | peripheral |
| Bpet3739 t*cbA*b | GI5 | chlorobenzene dioxygenase beta-subunit |
| Bpet3740 *tcbA*c | GI5 | chlorobenzene dioxygenase, ferredoxin subunit |
| Bpet3741 *tcbA*d | GI5 | chlorobenzene dioxygenase, NADH-ferredoxin reductase |
| Bpet3742 *tcbB* | GI5 | cis-chlorobenzene dihydrodiol dehydrogenase |
| Bpet3743 | GI5 | ring hydroxylating dioxygenase alpha subunit |
| Bpet3744 | GI5 | ring hydroxylating dioxygenase beta subunit |
|  |  |  |  |  |
| Bpet3747 *tcbR* | GI5 | transcriptional regulator | chlorocatechol | central |
| Bpet3748 *tcbC* | GI5 | chlorocatechol 1,2-dioxygenase |
| Bpet3749 *tcbD* | GI5 | chloromuconate cycloisomerase |
| Bpet3750 *bug77* | GI5 | solute receptor |
| Bpet3751 *tcbE* | GI5 | dienelactone hydrolase |
| Bpet3752 *tcbF* | GI5 | maleylacetate reductase |
|  |  |  |  |  |
| Bpet4008 *mhaA* | - | 3-hydroxyphenylacetate hydroxylase large component | 3-hydroxyphenylacetate | peripheral |
|  |  |  |  |  |
| Bpet4015 | - | LysR-family transcriptional regulator | homogentisate | central |
| Bpet4016 *hmgA* | - | homogentisate 1,2-dioxygenase |
| Bpet4017 *hmgB* | - | fumarylacetoacetase |
|  |  |  |  |  |
| Bpet4463 *kynU* | - | kynureninase | tryptophan | peripheral |
|  |  |  |  |  |
| Bpet4527 *pcaR* | - | transcriptional regulator |  |  |
| Bpet4528 *pcaI*4 | - | 3-oxoadipate CoA-transferase subunit A | catechol | central |
| Bpet4529 *pcaJ*4 | - | 3-oxoadipate CoA-transferase subunit B |  |  |
| Bpet4530 c*atC*2 | - | muconolactone isomerase |  |  |
| Bpet4531 | - | conserved hypothetical protein |  |  |
| Bpet4532 *catD*2 | - | 3-oxoadipate enol-lactone hydrolase |  |  |
|  |  |  |  |  |
| Bpet4737 | - | putative phenylacetyl-CoA ligase | unknown | peripheral |
