# Supplementary material for: The missing link: Bordetella petrii is endowed with both the metabolic versatility of environmental bacteria and virulence traits of pathogenic Bordetellae
Source: BMC Genomics. 2008 Sep 30;9:449. doi: 10.1186/1471-2164-9-449 (PMC2572626; doi:10.1186/1471-2164-9-449)
Supplement: Additional file 5 — Legends of the additional figures. [file 1471-2164-9-449-S5.doc]

**Additional Figure 1:**

**Dendrogramm showing the phylogenetic relationship of members of the genera *Bordetella*, *Achromobacter* and *Alcaligenes***

Strains of the various genera for which nearly complete 16S rDNA sequences are available were used for the calculations. *Cupriavidus necator* ATCC 43291 was taken as representative out-group. The tree was constructed using the neighbor-joining method [1]. The reliability of the inferred trees was tested by bootstrap analysis using 100 resamplings. Bootstrap values are denoted at the branching points. The scale bar indicates 0.01 nucleotide substitutions per position. Genus abbreviations used: *Bordetella* (B.), *Achromobacter* (Ac.) and *Alcaligenes* (Al.). GenBank sequence accession numbers used for this analysis are shown after the name of the bacterial strain. Type strains are indicated as (T). Completely genome sequenced *Bordetella* strains are boxed. The phylogenetic analysis was conducted using the MEGA4 program [2].

**Additional Figure 2:**

**Comparison of four *B*. *petrii* GIs and the *clc* element from *Pseudomonas* sp. strain B13**

From top to bottom: the *clc* element, GI3, GI1, GI2 and GI6. Only the regions of homology among the various GIs are shown; broken lines indicate that the respective GI continues without significant homology to the other islands. The length of the individual GIs is shown in parenthesis. While GI3 has a very similar size as the *clc* element, the other GIs are substantially larger. Gray bars represent the forward and reverse strands of DNA. The light blue boxes within the gray bars show the CDSs present on the GIs. The red blocks between the GIs represent regions of similarity, which were generated by TBLASTX; the darker the color, the higher the protein similarity. GI3 is most similar to the *clc* element. The display was generated by using the Artemis comparison software (ACT, http://www.sanger.ac.uk/Software/ACT/).

**Additional Figure 3:**

**Schematic presentation of the Bvg-systems of *B. pertussis* and *B. petrii***

On top, the structure of the BvgAS system of *B. pertussis* is shown and the signal induced phosphorelay is indicated. On the bottom, the domain structure of the *B. petrii* Bvg-related proteins is shown. Homologous domains are shown in identical colors.

**Additional References:**

1. Saitou N, Nei M: **The neighbor-joining method: a new method for reconstructing phylogenetic trees.** *Mol Biol Evol* 1987 **4:** 406-425.
2. Tamura K, Dudley J, Nei M, Kumar S: **MEGA4: Molecular evolutionary genetics analysis (MEGA) software version 4.0.** *Mol Biol Evol* 2007 **24:** 1596-1599.
